# Editorial: Impact and control of food- and waterborne protozoan parasites

**DOI:** 10.3389/fmicb.2024.1466883

**Published:** 2024-08-06

**Authors:** Olga Matos, Panagiotis Karanis, Romy Razakandrainibe

**Affiliations:** ^1^Global Health and Tropical Medicine, Instituto de Higiene e Medicina Tropical, Universidade Nova de Lisboa, Lisboa, Portugal; ^2^Environmental Health Institute, Faculdade de Medicina, Universidade de Lisboa, Lisboa, Portugal; ^3^Department of Basic and Clinical Sciences, University of Nicosia School of Medicine, Medical School, Nicosia, Cyprus; ^4^Université de Rouen Normandie, Laboratory of Parasitology and Mycology, UR7510 ESCAPE, University Hospital of Rouen, Rouen, France

**Keywords:** foodborne pathogens, protozoa, foodborne parasites, waterborne, contamination

## Protozoan parasites: the invisible menace

The rise of food—and waterborne diseases caused by protozoan parasites, including *Cryptosporidium, Cyclospora, Giardia*, and *Toxoplasma gondii*, increasingly threatens food safety.

*Cyclospora* is a causative agent of gastrointestinal outbreaks, primarily associated with fresh produce such as soft fruits and leafy vegetables. Also, the contamination of vegetables and fruits with *Cryptosporidium* has been reported in many countries worldwide. In addition, while infection with *Toxoplasma gondii* is usually mild or asymptomatic, this can present a serious health problem in immunocompromised persons and infections in pregnant women can present a severe risk to the baby. *T. gondii* can be transmitted to humans by consuming water and/or fresh vegetables and fruit contaminated with oocysts and raw or undercooked meat infected with tissue cysts. These parasites pose a significant public health hazard, demanding urgent attention from the scientific community and the food industry. A comprehensive, multifaceted approach is essential to safeguard our food supply and protect vulnerable populations.

In their paper titled “*Prevalence and risk factors associated with gastrointestinal parasites in goats (Capra hircus) and sheep (Ovis aries) from three provinces (Jiangsu, Shaanxi and Hunan) of China*,” Cai et al. discuss significant shifts in food consumption patterns in China. One notable change is the consumers' growing popularity of goats and sheep due to their low fat and cholesterol content, high protein, and appealing flavor. Gastrointestinal (GI) parasites infecting goats and sheep include helminths and protozoa, among which nematodes and coccidia are the most common.

Although the species *Cryptosporidium, Giardia*, and *Toxoplasma* were not investigated in the study, it was shown that the protozoan *Eimeria* was detected in a large majority of the samples (*n* = 1,081), accounting for 71.0% (767/1,081). The prevalence of helminths was 56.2% (607/1,081). The dominant species were *E. alijevi* in goats (67.3%, 562/835) and *E. parva* in sheep (30.1%, 74/246). The ground feeding mode, autumn season, and regions were relevant risk factors that significantly influenced the occurrence of GI parasites in goats and sheep. Investigating these GI parasites is pivotal, as it contributes to developing prevention strategies to minimize economic losses in small ruminant production and mitigate zoonotic parasite infections in humans.

## Detection and prevention: a scientific imperative

There is a critical need for rapid and standardized detection methods to address the growing threat of protozoan parasites in our food supply. Advances in molecular biology and diagnostic technologies offer promising avenues for more accurate and timely identification of these pathogens. Research into the parasitic load and prevalence in various foodstuffs, including fresh produce, meat, fish, and shellfish, is vital for understanding the scope of contamination and developing targeted interventions.

In their study titled “*First application of a droplet digital PCR to detect Toxoplasma gondii in mussels*”, Mancusi et al. applied and validated a droplet digital polymerase chain reaction (ddPCR) protocol on mussels to obtain a more sensitive diagnostic tool to detect and quantify *T. gondii* DNA. Bivalve molluscs, such as clams, cockles, mussels, and oysters, can filter significant quantities of water, thereby accumulating chemical and biological contaminants, including *T. gondii* oocysts. Furthermore, these molluscs can excrete the parasite in their feces several days after ingestion. Consequently, consuming raw or undercooked bivalve molluscs represents a substantial risk to human health. The ddPCR targeting the 529 bp repeated element gene of *T. gondii* demonstrated efficient DNA amplification of up to 8 genomic copies/μL. Analysis of the ddPCR data revealed distinct segregation between negative and positive droplets with minimal interface droplets, thus underscoring the technique's elevated specificity and efficacy. The ddPCR exhibited 100% sensitivity and specificity (95% confidence interval = 94.3–99.9). This study reported no significant intra-laboratory variance in results. The ddPCR holds promise for prompt, sensitive detection of low DNA concentrations of *T. gondii*, thus rendering it suitable for standardized food inspection across diverse matrices and mitigating the public health risks associated with this parasite.

In humans, the first-line point-of-care (POC) commercial tests for *Toxoplasma* are designated as *Toxoplasma* ICT IgG-IgM. These tests integrate the tachyzoites total lysate antigen (TLA) derived from mouse proliferation or *in vitro* tissue cultures. Although these tests provide 100% sensitivity and specificity in the USA, they have shown different sensitivity and specificity rates of 97 and 96%, respectively, in France, as per Chapey et al. (2017). Many efforts have been made to replace TLA with recombinant antigens (rAgs) to diagnose toxoplasmosis in humans and animals.

In commercial diagnostic test kits, the detection of IgG demonstrates high specificity and sensitivity, whereas IgM detection exhibits lower sensitivity (Khan and Noordin, 2020). The scientific community is actively involved in identifying novel antigens targeting IgM to enhance early infection diagnostics. This collective effort could pave the way for developing more performant tools. Nguyen et al., in their paper “*Identification of novel biomarkers for anti-Toxoplasma gondii IgM detection and the potential application in rapid diagnostic fluorescent tests*,” identified three novel antigens—EF1γ, PGKI, and GAP50—that specifically target IgM. Of the three antigens examined, GAP50 exhibited higher sensitivity in detecting IgM in rodent samples when used to fabricate a rapid strip test coupled with a fluorochrome (FITC) compared to TLA-based ELISA. The authors emphasize the distinctive immunoreactivity of GAP50, emphasizing its potential as a specific diagnostic biomarker to augment the sensitivity of FITC in IgM detection. The authors propose its suitability as a candidate antigen for integration into POC testing to detect IgM associated with *T. gondii* infections in patient samples.

The last study was titled “*Development of a targeted amplicon sequencing method for genotyping Cyclospora cayetanensis from fresh produce and clinical samples with enhanced genomic resolution and sensitivity*”. Leonard et al. addresses the issue of *Cyclospora cayetanensis*, for which no tools are currently available for genotyping contaminated fresh produce or environmental samples despite the increasing number of cyclosporiasis cases. The Centers for Disease Control and Prevention (CDC) employ eight markers for multi-locus sequencing typing (MLST), which involve individual conventional PCRs for each marker: CDS1, CDS2, CDS3, CDS4, HC378, HC360i2, MSR, and MT-junction. To achieve successful genetic clustering, genotyping data for a sample must be available for at least five markers or the three specific markers—HC378, HC360i2, and MSR, as per Nascimento et al. (2020). Leonard et al. developed a targeted amplicon sequencing (TAS) assay, combined with a bait-capture technique, to achieve the necessary sensitivity for genotyping *C. cayetanensis* in contaminated fresh produce samples. This TAS assay targets 52 loci, 49 located in the nuclear genome, and covers 396 currently known single nucleotide polymorphism (SNP) sites. The TAS assay facilitates the haplotyping of more markers, thereby capturing more significant genomic diversity from fecal samples with low parasite loads. It enhances genetic resolution and enables sequencing from artificially contaminated romaine lettuce and salad mix. Even at low contamination levels of 10 oocysts in 25 g of leafy greens, the assay can yield sequences for a minimum of 24 markers. As a molecular surveillance tool, this method may investigate the dispersion of *C. cayetanensis* in various environments.

## A call to action

The ongoing battle against food- and waterborne protozoan parasites necessitates collaboration among researchers, policymakers, and industry stakeholders to develop and implement effective detection, prevention, and treatment methods. As our understanding of these parasites and their impact on food safety improves, it is crucial to translate scientific discoveries into practical solutions that can be readily applied across food production and distribution networks.

In summary, protozoan parasites are increasingly prevalent in our food and water sources, posing significant risks. We can mitigate these pathogenic threats through interdisciplinary cooperation and research investment. The time to act is now before these threats become overwhelming challenges.

## Author contributions

OM: Writing – review & editing, Visualization, Validation, Supervision. PK: Writing – review & editing, Visualization, Validation, Supervision. RR: Writing – review & editing, Writing – original draft, Visualization, Validation, Supervision.
